# Identification of a Novel Deep Intronic Variant by Whole Genome Sequencing Combined With RNA Sequencing in a Chinese Patient With Menkes Disease

**DOI:** 10.3389/fgene.2022.852764

**Published:** 2022-03-31

**Authors:** Xiufang Zhi, Qi Ai, Wenchao Sheng, Yuping Yu, Jianbo Shu, Changshun Yu, Xiaoli Yu, Dong Li, Chunquan Cai

**Affiliations:** ^1^ Graduate College of Tianjin Medical University, Tianjin, China; ^2^ Tianjin Children’s Hospital (Children’s Hospital of Tianjin University), Tianjin, China; ^3^ Key Laboratory of Cancer Prevention and Therapy, Department of Pediatric Oncology, National Clinical Research Center for Cancer, Tianjin’s Clinical Research Center for Cancer, Tianjin Medical University Cancer Institute and Hospital, Tianjin Medical University, Tianjin, China; ^4^ Department of Hematology and Oncology, Tianjin Children’s Hospital, Tianjin, China; ^5^ Tianjin Pediatric Research Institute, Tianjin, China; ^6^ Tianjin Key Laboratory of Birth Defects for Prevention and Treatment, Tianjin, China; ^7^ Tianjin Kingmed Center for Clinical Laboratory, Tianjin, China; ^8^ Department of Neurology, Tianjin Children’s Hospital, Tianjin, China

**Keywords:** ATP7A gene, Menkes disease, whole genome sequencing, RNA sequencing, deep intronic variants

## Abstract

**Background:** Menkes disease (MD) is a rare X-linked connective tissue disorder of copper metabolism caused by pathogenic variant(s) in *ATP7A* gene. The aim of the present study is to determine the clinical characteristics and molecular basis of one patient with MD.

**Methods:** One 10-month-old Chinese boy who met the clinical manifestations of MD was enrolled in this study. Whole genome sequencing (WGS) was performed in the patient in order to identify the variant(s), followed by Sanger sequencing. RNA sequencing (RNA-seq) from whole blood was subsequently applied to assess the effect of variant on transcription levels, and reverse transcriptase-polymerase chain reaction (RT-PCR) was performed for further validation. In addition, X chromosome inactivation (XCI) status of the patient’s mother at the DNA level was measured by capillary electrophoresis.

**Results:** The patient suffered from intermittent convulsions for more than 6 months, with psychomoto retardation and neurodegenerations. The patient also had curly hair, hypopigmented skin, cutis laxa, decreased muscle strength and hypotonia. MRI showed the intracranial arteries were tortuous with some “spiral” changes. The patient’s serum ceruloplasmin level was low. WGS revealed one novel hemizygous variant, c.2627-501C > T (NM_000,052.7), located in the deep intronic sequence of *ATP7A* gene. Sanger sequencing confirmed that the variant was inherited from his mother. RNA-seq confirmed the variant itself, and identified a pseudo-exon inserted between exons 12 and 13 in mRNA of *ATP7A*. The sequencing results of RT-PCR from the patient confirmed this finding, while neither of his parents detected aberrant splicing. The Capillary electrophoresis results showed that the patient’s mother had a skewed XCI.

**Conclusion:** Our finding of the variant enlarges the variant spectrum in the *ATP7A* gene. This is a novel deep intronic variant which leads to the activation of a pseudo-exons in the *ATP7A* gene, and it demonstrates the usefulness of WGS combined with RNA-seq, in terms of revealing disease-causing variants in non-coding regions. Furthermore, the fact that the deep intronic variants cause disease by the activation of pseudo-exon inclusion indicates that in MD this might be an important mechanism.

## Introduction

MD, also known as Menkes kinky hair syndrome, is a rare X-linked recessive genetic disease of impaired copper metabolism caused by variants in *ATP7A* gene (Caicedo-Herrera et al., 2018; Patel et al., 2017). Clinical manifestations include neurological symptoms and systemic multi-system dysfunction (Altarelli et al., 2019). According to clinical manifestations and progression, it can be divided into three subtypes: 1) classical type accounts for 90–95%, manifested as progressive neurodegeneration, seizures, connective tissue abnormalities and “kinky” with hair, and ultimately death in early childhood; 2) atypical MD has various clinical phenotypes of the classical type, but the degree is milder and the survival time is longer; and 3) occipital horn syndrome only affects connective tissue and bones, with the mildest form (Bonati et al., 2017; Kaler and Distasio, 1993; Kodama et al., 2012; Smpokou et al., 2015).

*ATP7A* gene, located on the Xq13.3 chromosomal region, contains 23 exons and encodes the transmembrane P-type ATP enzyme which can transport copper to the extracellular or Golgi apparatus by phosphorylation/dephosphorylation (Hartwig et al., 2019; Horn and Wittung-Stafshede, 2021; Skjorringe et al., 2017). Variants in *ATP7A* gene cause the inactivation of copper transport ATP enzyme, which block the absorption of copper in intestinal mucosa and the transport of copper across blood-brain barrier, ultimately leading to nervous system abnormalities and multi-system dysfunction (Chen et al., 2020; Maung et al., 2021; Petris et al., 2000; Prithvi et al., 2019). MD is mostly caused by nonsense variants, frame shift variants and splice site variants (Cao et al., 2017; de Gemmis et al., 2017; Kaler et al., 2020; Tumer, 2013). About 22% of *ATP7A* variants are splice site variants (Moller et al., 2009). Splice site variants may cause one or more exons complete skipping or retention of introns by activation of cryptic splice sites within exons or introns (Anna and Monika, 2018; Parada et al., 2014), and eventually affect the structure and function of transmembrane P-type ATP enzyme or reduce the expression of normal products (Moller et al., 2000). In the clinically discovered *ATP7A* variant-related MD patients, the vast majority of splice site variants occur in the intrinsic splicing zone (Skjorringe et al., 2011). The pathogenesis of deep intronic variants is rarely reported.

One possible important reason why deep intronic variants are always overlooked is that the detection methods are inappropriate. As an important means of identify disease-causing genetic variants of individuals affected with a genetically undiagnosed rare disorder, the main disadvantage of whole exome sequencing (WES) is that it cannot indicate disease-causing variants in non-coding regions. WGS in principle reveals all genetic variants, however, the main challenges are the sizeable variants number and poor interpretation of the non-coding variants. RNA-seq can directly detect gene expression, which help to reveal splice defects, the mono-allelic expression of heterozygous loss-of-function variants, and expression outliers, thereby providing functional data to support the clinical interpretation of variants (Cummings et al., 2017; Kremer et al., 2017). Therefore, the combination of WGS and RNA-seq displays strong superiority in the detection and analysis of non-coding variants.

In the present study, we identified a novel hemizygous variant of *ATP7A* located in deep intronic sequences by WGS combined with RNA-seq in a Chinese family. In addition, we described the clinical profile, laboratory and radiological findings of the patient and performed a variant analysis of *ATP7A* in the patient with MD.

### Patients, Materials and Methods

#### Ethics Statement

The study was approved by the Ethics Committee of Tianjin Children’s Hospital (Tianjin University Children’s Hospital), and the study approval reference number was 2016021. Written informed consent of the patient was obtained from his parents, and the parents gave their own written informed consent to take part in this study. All study procedures adhered to the tenets of the Declaration of Helsinki.

#### Patients

The patient, a 10-month-old Chinese boy with non-consanguineous parents, was admitted to Tianjin Children’s Hospital because of convulsions. All of the patient’s clinical information was collected from medical records. The peripheral blood samples were collected from the patient and his parents. All the experiments performed with human materials were in accordance with the relevant guidelines and regulations.

#### DNA and RNA Isolation

Genomic DNA was isolated from peripheral venous blood of the patient and both parents using Blood Genomic DNA Mini Kit (CWBIO, Jiangsu, China) according to the manufacturer’s protocol. Total RNA was extracted from peripheral venous blood using Lymphocyte Separation Medium (SOLARBIO SCIENCE &TECHNOLOGY CO., LTD., Beijing, China) and Trizol reagent (TIANGEN BIOTECH CO., LTD., Beijing, China). DNA and RNA was quantified by Nanodrop Spectrophotometer (THERMO FISHER SCIENTIC, Shanghai, China). DNA and blood samples were stored at −20°C. RNA was stored at −80°C.

### WGS and in Silico Analysis of Potential Splice Variant

WGS was performed by KingMed company (Guangzhou, China) on the patient. The DNA was performed with 150-bp paired-end sequencing and average coverage of 50-fold in more than 95% of the target regions, including the coding region and intronic region. WGS data were processed following the standard procedure. Low-quality reads, adapters, and sequences with more than 5% unknown bases were removed from the raw reads. Sequenced reads were aligned to the human reference genome hg19 using the Burrows-Wheeler Aligner software. Duplications were marked with Picard and BAM was sorted with Samtools2. GATK Best Practices Pipeline was used in the process of SNP and INDEL calling. SNPs and INDELs were annotated using Annovar and local software. The HGMD, 1,000 genomes, OMIM, dbSNP, Clinvar and local database were used for variants annotation. Specifically, all the variants were firstly filtered using the dbSNP, 1,000 genomes, and local databases, deleting all variants with frequency >1% in the population. All the remaining filtered variants were searched in the OMIM and HGMD databases for identifying the variants of genes associated with the disease phenotype. Then, the variant pathogenicity classification followed the variant classification criteria formulated by American College of Medical Genetics and Genomics (ACMG) and the Association for Molecular Pathology (AMP) (Richards et al., 2015; Zhang et al., 2020). Clinical significance grading was done as described previously (Cai et al., 2021). Sanger sequencing was performed for the candidate variant(s) in the patient and his parents for confirmation.

To analyze the effect of the candidate variant(s) on pre-mRNA splicing, we performed *in silico* analysis using several different freely available software including NetGene2 (http://www.cbs.dtu.dk/services/NetGene2/), Splice Site Prediction by Neural Network (www.fruitfly.org/seq_tools/splice.html), and ASSP model (wangcomputing.com/assp). The variant(s) designations were based on *ATP7A* gene (NM_000,052.7) from NCBI, and the gene sequence is from NC_000,023.11. We analyzed the new splice site and its effect on splicing by comparing the difference of the splice site between the wild type and the variant(s).

### RNA-Seq

RNA-seq assay was applied to confirm the splicing effect of the candidate variant(s). Whole-blood samples were collected in PAXgene whole blood RNA tubes (BD), and intracellular RNA was extracted using PAXgene blood RNA kit (QIAGEN) according to the manufacturer’s recommendation. RNA was quantified and then processed to sequencing library preparation using Ribo-off rRNA depletion kit (human/mouse/rat) and VAHTS^®^ universal V8 RNA-seq library prep kit for Illumina (Vazyme). The qualified library was sequenced on an Illumina Novaseq 6,000 plateform with PE150 mode at the Tianjin KingMed Center for Clinical Laboratory for Translational Genomics, and approximately 30–50 million raw reads were expected to be generated.

Raw reads were processed by trimming poly-A/T stretches and Illumina adapters using Cutadapt software and resulting reads shorter than 30bp were discarded. Sequencing data was mapped to the GRCh37 reference genome using STAR and HISAT2 software with default parameters. Transcripts were assembled by Stringtie software from the reads alignment data for abnormal transcripts detection. The iGV v2.9.4 software was applied for manual analysis for the genomic regions of interest.

### RT-PCR

Complementary DNAs (cDNA) were synthesized with the FastKing RT Kit (With gDNase) (TIANGEN BIOTECH CO.,LTD., Beijing, China) according to the manufacturer’s instruction. To determine the consequence of the variant for the *ATP7A* gene transcript, we performed PCR experiments on cDNA using primers located in exon 9 (Forward, 5′-CTT​CTA​TGT​TCC​TGG​AGC​G-3′) and in exon 15 (Reverse, 5′-ATC​GTT​TCT​GTT​CGG​GAG-3′). Due to the low expression of *ATP7A* in whole blood, we performed two sequential amplification reactions, and both of which used the same primers. Alternatively, the product of the first amplification reaction was used as the template for the second PCR, which was amplified by the same primers as the first primer pair. The final PCR products from cDNA was resolved by 1.5% agarose gel electrophoresis and sequenced (GENEWIZ, Hangzhou, China).

### XCI Status Measurement by the Human Androgen Receptor (HUMARA) Assay

The XCI status at the DNA level was measured using HUMARA assay based on the highly polymorphic CAG trinucleotide repeat in the promoter area of the androgen receptor (AR). Specifically, genomic DNA was digested with methylation-sensitive restriction endonuclease HhaI (TAKARA BIO, Japan). The CAG repeat regions from digested and undigested DNA samples were amplified by PCR with forward primer (GCT​GTG​AAG​GTT​GCT​GTT​CCT​CAT) and reverse primer (TCCAGAATCTGTTC CAGAGCGTGC) (Pan et al., 2021), then the PCR products were resolved by 1.5% agarose gel electrophoresis to confirm that DNA had been fully digested. Additionally, the same DNA samples were amplified by PCR using same primers but the forward primer was end-labeled with FAM fluorescence. The products were analysed by capillary electrophoresis (BECKMAN COULTER, United States). The proportion of variant allele on the inactive X chromosome was calculated according to the standard formula (Thouin et al., 2003). XCI status was classified as random (XCI<70%), or skewed (XCI≥70%) (Pu et al., 2010; Sato et al., 2004). If the ratio was ≥90%, the XCI pattern was considered to be extremely skewed (Yoon et al., 2008).

## Results

### Clinical Characteristics of the Patient

The male Chinese patient (proband) was born at term after an uncomplicated pregnancy, with third degree contamination of amniotic fluid, and neonatal jaundice after birth. His birth weight was 2.25 kg. Convulsions were seen as early as 4 months after birth, the patient presented with right-sided eyelid myoclonus for several seconds over 10 times a day. The second seizure type appeared at 6 months of age. The patient showed flexor spasms in either a isolation seizure or a cluster of seizures. When a cluster of seizures occurred, seizure occurred 2–8 times per day and the maximum duration was about 20 s. At 8 months of age, the third type, generalized tonic-clonic seizures, occurred. The patient received 7–8 times of seizure per day, each lasting for 30 s to 1 min. There were no loss of consciousness in the first two types of seizure. Only spasms were described in the last 1 month with an exacerbation of symptoms. The patient received antiepileptic therapy with topiramate and levetiracetam, but the effect was poor. At present, the patient weighed 7.1 kg. Developmental milestones of the patient was normal before 4 months of age. However, developmental regression was beginning to appear at 4 months of age. The age of the patient was10.6 months old at this visit. He could not raise his head, turn over, or sit alone. His deciduous teeth had not erupted yet. He could not make a laugh and sought to track parallax. He could only eat with a dropper at presentation, showing global developmental delay.

On physical examination, his head was flat with irregular shape. The hair was sparse, short, and curly while the skin was fair, rough, and loose. One 3 mm × 3 mm café au lait spot could be seen on the inside of the right upper arm. He had pectus excavatum, decreased muscle strength, hypotonia and decreased muscle volume.

**FIGURE 1:**
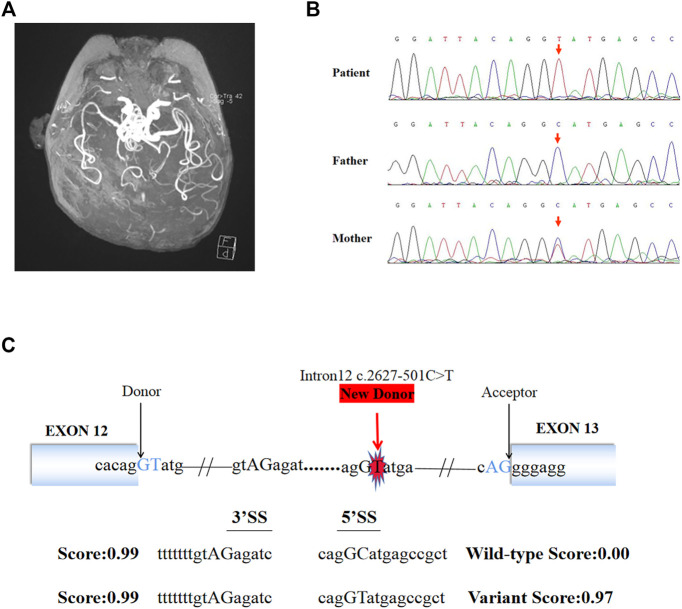
Brain MRI, Sanger sequencing results and splice site prediction schematic of patient. **(A)** Magnetic resonance angiogram showed the intracranial arteries were tortuous with some “spiral” changes. **(B)** Sanger sequencing confirmed that the variant (c.2627-501C > T) of *ATP7A* gene was inherited from the patient’s mother. **(C)** The prediction results *in silico* showed the existence of a new 5′ donor splice site and a cryptic 3′ receptor splice sites. The boxes represented exons, the black arrows pointed to the normal classic splice site, and the red arrow pointed to the new donor splice site caused by the variant.

Video EEG showed hypsarrhythmia. Brain MRI showed patchy flaky slightly long T2 signal in the white matter of bilateral frontal, temporal, and parietal cortices. DWI showed a small patch hyperintensity in the bilateral centrum semiovale. Moreover, MRI showed widened ventricles, sulci and cisterns. Brain MRA showed the intracranial arteries were tortuous with some “spiral” changes (Figure 1A). The serum ceruloplasmin was 0.05 g/L (normal range 0.15–0.30 g/L). MD was suspected clinically. Given the severe neurological symptoms, it was considered to be the classical type. An extensive previous genetic workup, including karyotype, WES, trio WES, were negative.

### Identification and Analysis of the Candidate Variant

140G sequencing data was acquired after WGS for the proband and 40.5 million SNVs, 931 thousands indels, and no pathogenic CNVs were detected in the proband’s WGS data. After variant pathogenicity classification and clinical significance grading screening process, only one candidate variant, NC_000,023.10: g.77275240 C > T (hg37, chrX), c.2627-501C > T (NM_000,052.7), located in deep intron 12 of *ATP7A* gene was identified in the sequencing data as a potential causative variant for the proband’s phenotype. Sanger sequencing confirmed that the c.2627-501C > T variant was inherited from the mother carrying heterozygous variant (Figure 1B). This variant has never been reported in ClinVar database, HGMD database, or gnomAD database before. We submitted the sequence of the interest region to splice site predictor programs (NetGene2, Splice Site Prediction by Neural Network, ASSP model) and all of them confirmed that the variant activated a new splice donor site (Figure 1C).

### Aberrant Splicing Caused by the Varant in Transcription Level

**FIGURE 2:**
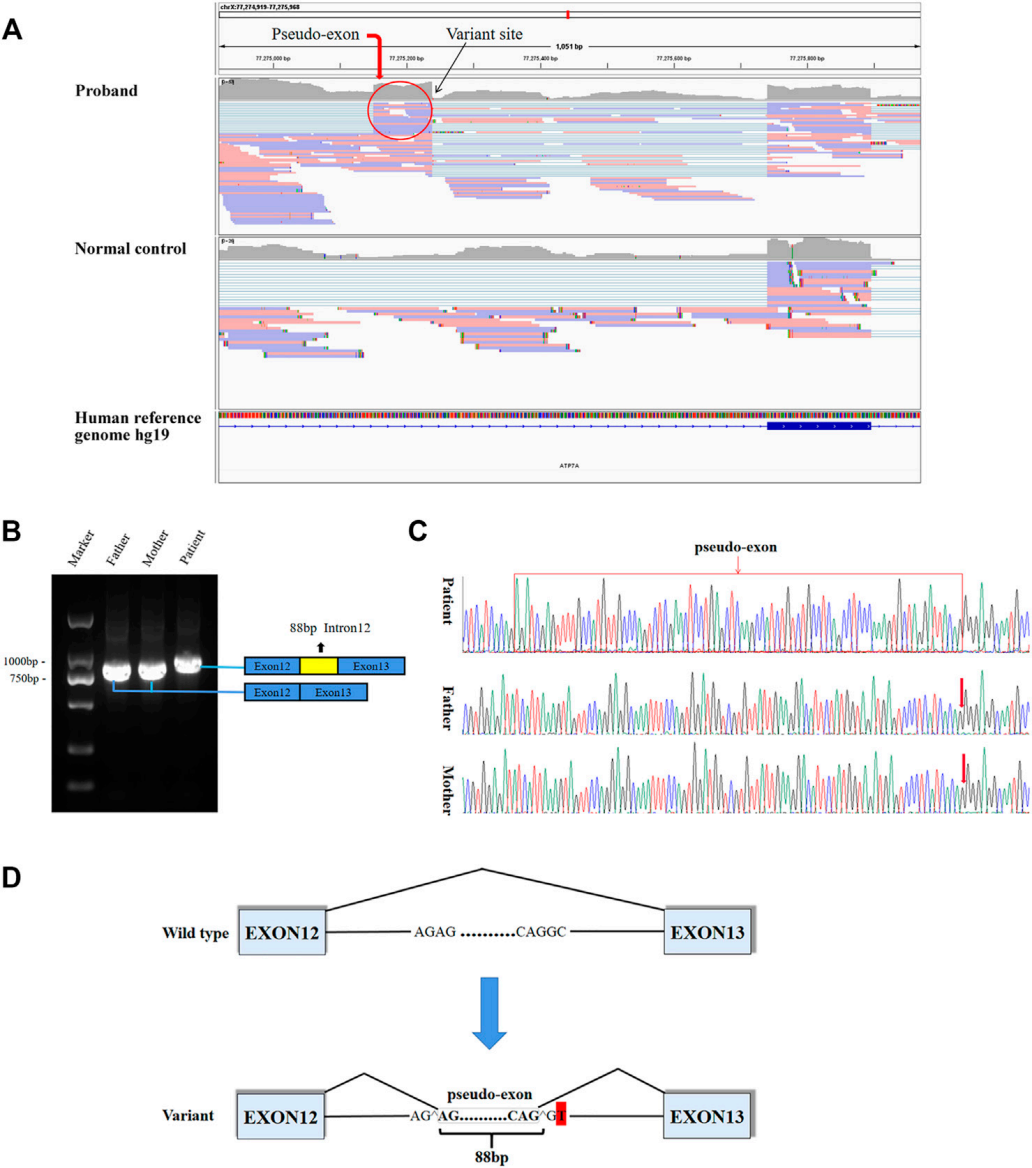
Aberrant splicing caused by the varant. **(A)** RNA-seq demonstrated an abnormal splicing pattern that resulted in an out-of-frame pseudo-exon between exons 12 and 13. The black arrow pointed to the variant site, and the red circle was the fragment from intron 12 inserted between exons 12 and 13. **(B)** In agarose gel electrophoresis of RT-PCR, a lager product size in patient compared with the normal size of his parents. The blue boxes represented exons, and the red box represented intron fragment inserted between exons 12 and 13. **(C)** The sequencing results of RT-PCR showed there was a 88bp pseudo-exon fragment between exons 12 and 13 in mature ATP7A mRNA of the patient compared to his normal parents. **(D)** Schematic diagram of WT and variant splicing patterns showed that the variant site created a new 5′ donor splice site and activated the upstream 3′ acceptor splice site concomitantly, thereby creating the aberrant splicing. Boxes represented exons. Straight lines represented base sequences. Broken lines represented splicing modes.

8.8G sequencing data was acquired after RNA-seq for the proband. The *ATP7A* gene region was carefully looked through manually using the iGV software and we successfully detected the c.2627-501C > T variant. The data also demonstrated clearly an abnormal splicing pattern that resulted in an out-of-frame pseudo-exon between exons 12 and 13 (Figure 2A) which predicted to cause frame shift and protein truncation (p. Gly876Cysfs*26). By the way, the sequencing depth for the c.2627-501C site was only 5x and variants with such low depth were rendered as too much low quality data (although the C > T mutation frequency was 5/5) and were filtered after variant calling with default parameters. For further verification of the impact of the novel variant on splice site, we performed RT-PCR. In agarose gel electrophoresis of RT-PCR, cDNA analysis revealed a lager product size in patient compared with the normal size of his parents (Figure 2B). Direct sequencing of the patient’s amplified fragment revealed aberrant RNA splicing effect between exons 12 and 13, which resulted in the appearance of a pseudo-exon in the splicing process (Figure 2C), which was consistent with the RNA-seq result in terms of variant analysis. Of note, only wild-type (WT) *ATP7A* mRNA expression was detected in both electrophoresis and direct sequencing in the patient’s mother.

### Pathogenic Classification of the Variant

According to the ACMG/AMP variant classification criteria, the c.2627-501C > T variant could be classified as ‘likely pathogenic’ (PP4-Moderate, PS3-Moderate, PM2-Supporting, PP3-Supporting) (Richards et al., 2015; Zhang et al., 2020).

### Evaluation of XCI

**FIGURE 3:**
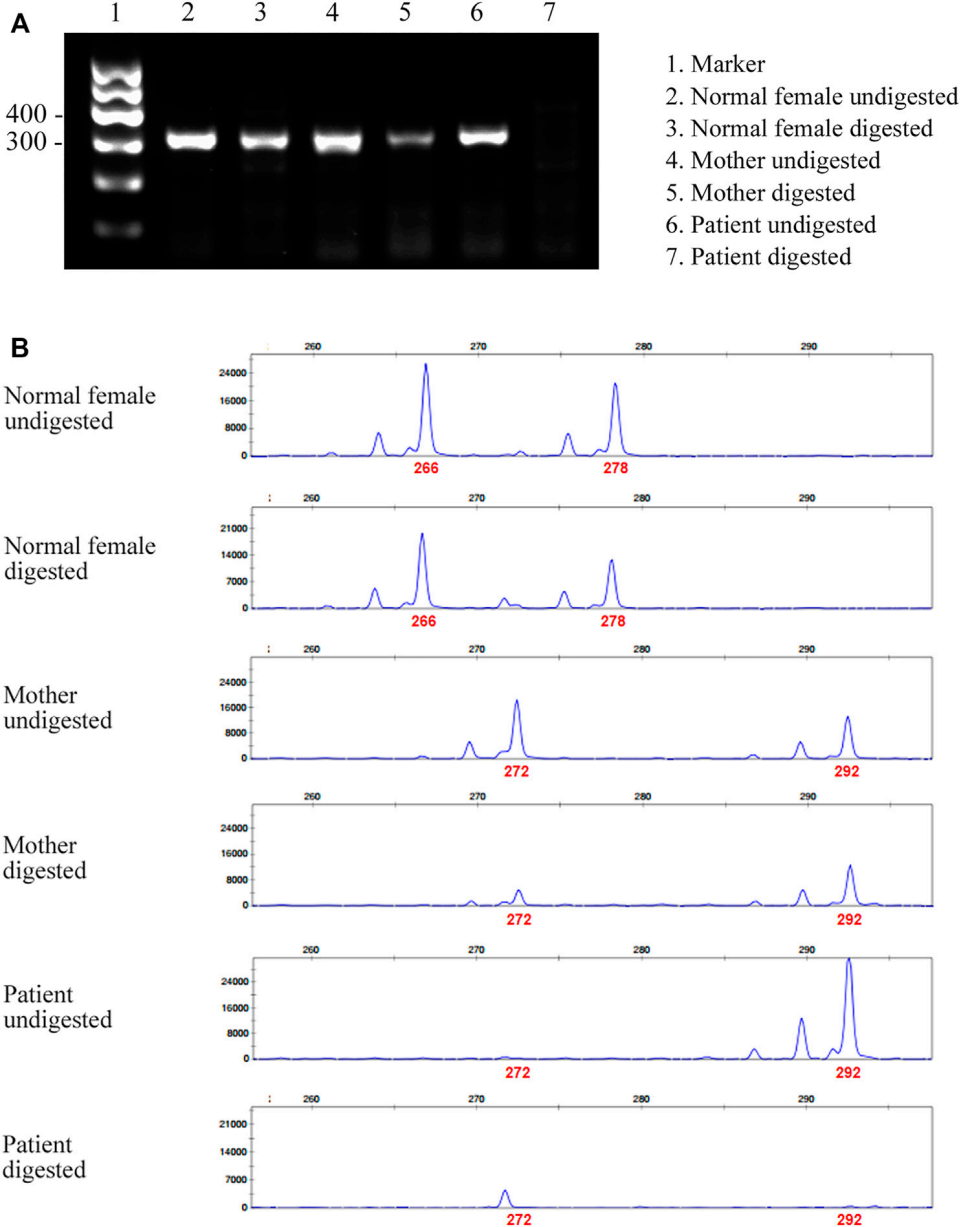
Agarose gel electrophoresis and capillary electrophoresis of XCI. **(A)** The result of agarose gel electrophoresis showed that there was no amplification band in the patient’s and his father’s DNA after digestion, indicating that the DNA was completely digested. **(B)** The result of capillary electrophoresis showed that there was one patient’s product with a size of 292 bp before DNA digestion, suggesting that this was the X chromosome carrying the variant. There were two mother’s products, 272 bp (WT) and 292 bp (variant), both in digested DNA and undigested DNA. The result showed skewed pattern of XCI.

Given that only WT *ATP7A* mRNA expression was detected in the patient’s mother, we performed the HUMARA assay for XCI on samples from the patient and his parents to evaluate the XCI state and to better understand the mechanism. The results of agarose gel electrophoresis showed that the DNA was completely digested (Figure 3A). The results of capillary electrophoresis showed that there was one patient’s product with a size of 292 bp before DNA digestion, suggesting that this was the X chromosome carrying the variant (Figure 3B). There were two mother’s products, 272 bp (WT) and 292 bp (variant), both in completely digested DNA and undigested DNA (Figure 3B). The proportion of variant allele on the inactive X chromosome was calculated as 78.0% indicating a skewed XCI model compare with 45.3% in normal female control.

## Discussion

In this study, the patient presented with epilepsy, rough and loose skin, sparse and curly hair, and psychomoto retardation. The level of serum ceruloplasmin was low. Otherwise, the patient’s MRI showed that the intracranial arteries were tortuous with some “spiral” changes. All the above manifestations were highly matched with MD. The complete diagnosis of MD still required genetic variant evidence. As a result, the patient and his parents underwent WES, trio WES in other medical institution, but no abnormality consistent with the patient’s clinical manifestations was found. Considering the limitations of WES including the inability to detect non-coding variants in intronic regions (Burdick et al., 2020; Jelin and Vora, 2018) and balanced translocation of chromosomes, we performed a karyotype analysis for the patient and the chromosomal analysis revealed a normal karyotype with 46, XY.

Due to these negative results and strong demands from the patient’s parents to find the etiology of the diease, we performed WGS for its capability to detect a full range of common and rare genetic variants of different types across almost the entire genome (Lappalainen et al., 2019). WGS found a deep intronic variant in the patient’s *ATP7A* gene (c.2627-501C > T). Then, the Sanger sequencing found that the variant was inherited from his mother.

It has been reported that deep intronic variant results in intron fragments (also known as cryptic exons or pseudo-exons) being inserted into mature transcripts, and leads to frame shift or premature termination of translation (Anna and Monika, 2018). Functionally, these variants create new acceptor or donor sites, which are recognized by the splicing complex and interact with existing intron cryptic splice sites. It is also possible that deep intronic variants lead to the generation of new regulatory elements (such as splicing enhancers) and the recognition of specific introns. To explore the effect of the variant on transcription level, we performed RNA-seq. RNA-seq can directly detect gene expression and provide functional data that supports clinical interpretation of variants. Although RNA-seq is a supplementary tool, its advantage lies in detecting the functional consequences of DNA variants that affect splicing and gene expression levels. Therefore, it supports the interpretation of variants of uncertain significance, and reorders variants that are ignored by DNA sequencing. The combination of RNA-seq with WES or WGS analysis may provide more information than WES or WGS analysis alone (Fresard et al., 2019; Gonorazky et al., 2019; Lee et al., 2020). In our case, based on the guidance of WGS to find the causative deep intronic variants, RNA-seq analysis found abnormal gene expression and splicing. RNA-seq result of the patient showed that a pseudo-exon (88bp) from intron 12 located between exons 12 and 13 was contained in the mature mRNA, and confirmed by RT-PCR. Due to the variant, a new donor splice site was generated, and the upstream cryptic splice acceptor site was activated, leading to 88 bp insertion from the new donor splice site upstream in intron 12 (Figure 2D). Finally, a pseudo-exon fragment from intron 12 was included in the mature mRNA, which resulted in a frame shift and a premature termination codon (p. Gly876Cysfs*26). A deep intron c.3718-2477C > T variant located in intron 19 of *CFTR* gene has been reported in the literature. This variant leads to the generation of a new donor site, resulting in an 84 bp pseudo-exon containing into the mature mRNA. This pseudo-exon contains an in-frame termination codon, so the translated protein is shorter and non-functional (Anczukow et al., 2012; Anna and Monika, 2018). However, there is a limitation in our study. At present, we cannot explain the mechanisms of how the newly formed donor splice site and cryptic acceptor splice site caused by the deep intronic variant lead to aberrant splicing. We will further verify these issues in follow-up research.

In this study, it was learned through WGS and Sanger sequencing that the patient’s variant was inherited from his mother, but the subsequent RT-PCR results showed that only WT *ATP7A* mRNA expression was detected in the patient’s mother, which was same with that of the father’s. We performed the HUMARA assay for XCI and the result suggested that the patient’s mother had a skewed XCI with a preferential inactivation of the X chromosome that carried the variant allele. This result may explain why the patient’s mother could only detect WT *ATP7A* mRNA expression.

Due to the high mortality rate of MD, patients with classic MD generally die before the age of three. Early diagnosis of MD and identification of pathogenic variants are the basis of genetic counseling. The results of this study are of great significance for this family’s genetic counseling, preventing the birth of children with MD (Elliott, 2020; Perge and Igaz, 2019; Yang and Kim, 2018). Early diagnosis may also be meaningful for MD gene therapy. It has been reported that Antisense oligonucleotides can improve the abnormal splicing effect of the aforementioned *BRCA2* gene intron 12, c.6937 + 594T > G to a certain extent (Anczukow et al., 2012). Gene therapy is still in the exploratory stage, however, it is expected that MD caused by such splice site variants can be treated by gene therapy in the future.

## Conclusion

Our finding of the variant enlarges the variant spectrum in the *ATP7A* gene. This is a novel deep intronic variant which leads to the activation of a pseudo-exons in the *ATP7A* gene, and it demonstrates the usefulness of WGS combined with RNA-seq, in terms of revealing disease-causing variants in non-coding regions. Furthermore, the fact that the deep intronic variants cause disease by the activation of pseudo-exon inclusion indicates that this might be an important mechanism in MD.

## Data Availability

All data relevant to the study are included in the article. The whole-genome sequencing data are not publicly available as these could compromise research participant privacy. Specific variant requests or other data are available from the corresponding author CC upon reasonable request.
